# Randomised feasibility study of prehospital recognition and antibiotics for emergency patients with sepsis (PhRASe)

**DOI:** 10.1038/s41598-021-97979-w

**Published:** 2021-09-20

**Authors:** Jenna Jones, Susan Allen, Jan Davies, Timothy Driscoll, Gemma Ellis, Greg Fegan, Theresa Foster, Nick Francis, Saiful Islam, Matt Morgan, Prabath W. B. Nanayakkara, Gavin D. Perkins, Alison Porter, Timothy Rainer, Samuel Ricketts, Bernadette Sewell, Tracy Shanahan, Fang Gao Smith, Michael A. Smyth, Helen Snooks, Chris Moore

**Affiliations:** 1 Swansea University Medical School, ILS2, Singleton Campus, Wales, SA2 8PP UK; 2 Cardiff and Vale University Health Board, Wales, UK; 3 Public Contributor, Wales, UK; 4 East of England Ambulance Service NHS Trust, Royston, England, UK; 5 University of Southampton, Southampton, UK; 6 Amsterdam University Medical Centre, Amsterdam, The Netherlands; 7 University of Warwick, Warwick, England, UK; 8 Welsh Ambulance Services NHS Trust, Wales, UK; 9 Swansea Centre for Health Economics, Swansea University, Wales, UK; 10 University of Birmingham, Birmingham, England, UK

**Keywords:** Clinical trials, Randomized controlled trials, Outcomes research, Health services

## Abstract

Severe sepsis is a time critical condition which is known to have a high mortality rate. Evidence suggests that early diagnosis and early administration of antibiotics can reduce morbidity and mortality from sepsis. The prehospital phase of emergency medical care may provide the earliest opportunity for identification of sepsis and delivery of life-saving treatment for patients. We aimed to assess the feasibility of (1) paramedics recognising and screening patients for severe sepsis, collecting blood cultures and administering intravenous antibiotics; and (2) trial methods in order to decide whether a fully-powered trial should be undertaken to determine safety and effectiveness of this intervention. Paramedics were trained in using a sepsis screening tool, aseptic blood culture collection and administration of intravenous antibiotics. If sepsis was suspected, paramedics randomly allocated patients to intervention or usual care using scratchcards. Patients were followed up at 90 days using linked anonymised data to capture length of hospital admission and mortality. We collected self-reported health-related quality of life at 90 days. We pre-specified criteria for deciding whether to progress to a fully-powered trial based on: recruitment of paramedics and patients; delivery of the intervention; retrieval of outcome data; safety; acceptability; and success of anonymised follow-up. Seventy-four of the 104 (71.2%) eligible paramedics agreed to take part and 54 completed their training (51.9%). Of 159 eligible patients, 146 (92%) were recognised as eligible by study paramedics, and 118 were randomised (74% of eligible patients, or 81% of those recognised as eligible). Four patients subsequently dissented to be included in the trial (3%), leaving 114 patients recruited to follow-up. All recruited patients were matched to routine data outcomes in the Secure Anonymised Information Linkage Databank. Ninety of the 114 (79%) recruited patients had sepsis or a likely bacterial infection recorded in ED. There was no evidence of any difference between groups in patient satisfaction, and no adverse reactions reported. There were no statistically significant differences between intervention and control groups in Serious Adverse Events (ICU admissions; deaths). This feasibility study met its pre-determined progression criteria; an application will therefore be prepared and submitted for funding for a fully-powered multi-centre randomised trial.

Trial registration: ISRCTN36856873 sought 16th May 2017; https://doi.org/10.1186/ISRCTN36856873

## Introduction

### Background

Sepsis is a life threatening condition which requires rapid recognition and treatment in order to not only prevent death, but also to minimise the damage that is does to a person’s body. Sepsis can lead to organ and limb damage, leaving patients with significant morbidity if they survive. Approximately 44,000 people die of sepsis every year in the UK^1^—more than are diagnosed with bowel cancer annually^2^.

There is evidence that administering antibiotics early reduces complications and death from sepsis^3–6^. It is known that recognition of sepsis by Emergency Medical Service (EMS) personnel in the prehospital phase of care leads to faster diagnosis and treatment in the Emergency Department (ED)^7,8^. Around half of patients with sepsis are conveyed to the ED by ambulance^9,10^, with an average prehospital care time of 45 min^11^, therefore, EMS personnel can play a significant role in the management of sepsis.

Early recognition and management of sepsis by EMS personnel offers a potentially valuable opportunity to deliver interventions early, but the evidence base to support the use of prehospital antibiotics is weak^12,13^. In 2016/17, when this feasibility study was first conceived, there was no well-defined prehospital protocol for management of sepsis in the UK. In 2019, the Joint Royal Colleges Ambulance Liaison Committee (JRCALC), issued a prehospital clinical practice guideline for the management of sepsis, which recognises the potential value of pre-hospital sepsis screening tools, but acknowledges that, to date, none have been validated for use^14^.

### Aim

To assess the feasibility of paramedics screening patients for severe sepsis, collecting blood cultures and administering IV antibiotics, and to assess trial methods to inform the development of a fully-powered randomised controlled trial (RCT), to test the clinical and cost-effectiveness of prehospital IV antibiotics, if indicated.

### Objectives

#### Intervention development

To work with clinicians, paramedics, pharmacists, and service users to develop a prehospital intervention for sepsis, comprising:

- Screening patients for sepsis
- Protocol for collection of blood cultures, administering IV antibiotics, and handover of care to ED
- Training for paramedics to deliver the agreed protocol

#### Intervention feasibility

To assess:

- paramedic uptake and satisfaction with the training package
- paramedic compliance with treatment protocol
- safety and acceptability of the intervention to patients and paramedics

#### RCT feasibility

To clarify primary and secondary outcome measures for full trial and assess:

- trial recruitment, randomisation, and data collection processes
- sample size requirements and attrition rates
- availability of outcome data

#### Full trial planning

To assess our findings against our progression criteria and, if met, draft a full trial protocol and application for funding.

## Methods

The protocol for this feasibility study has been published^15^. Ethical approval was granted by Wales Research Ethics Committee 4, reference 17/WA/0186, and the trial registered on the ISRCTN database (ISRCTN36856873) on 16/05/2017. Research has been conducted in accordance with the Declaration of Helsinki. The design of the trial is a single-centre randomised parallel-group feasibility trial, with 1:1 allocation ratio. Patients were eligible if they were adult (18 years or over), with severe sepsis (see additional file for our Sepsis Screening Tool), and attended by a study paramedic within the catchment area of the participating trial hospital. Patients were excluded if they were known or thought to be pregnant, or known to be allergic to antibiotics.

The study was conducted in the Cardiff & Vale of Glamorgan area of Wales, UK. Welsh Ambulance Service paramedics based at the five ambulance stations operating in the geographical catchment area of the University Hospital of Wales (UHW) were invited to take part. Data were collected from Patient Clinical Records (PCRs) completed by study paramedics, and supplemented by entries made in the PhRASe study logbook, which was secured in the resuscitation area of the ED.

### Interventions

If the patient was randomly allocated to usual care, the paramedic would provide the patient with oxygen to maintain saturations over 94% (88–92% if known to have chronic obstructive pulmonary disease) and administer 250 ml boluses of 0.9% sodium chloride (up to 2000 ml if the patient is hypotensive e.g. systolic blood pressure less than 100 mmHg). If the patient’s clinical condition was judged as serious or critical, paramedics could pre-alert the receiving hospital so that the patient could be taken directly to a resuscitation bay. If the patient was randomly allocated to experimental care, usual care would be supplemented by collection of blood cultures, and IV administration of 2 g cefotaxime. Participating paramedics were trained in use of this new protocol in small, face to face group sessions, conducted by the Paramedic Research Support Officer (PRSO).

### Patient consent

Given the potential for severe sepsis to impair mental capacity and the need to provide urgent treatment, in accordance with the Mental Capacity Act 2005, we sought approval from the Research Ethics Committee to enrol patients prior to obtaining written informed consent for the research^16^. At the time of paramedic attendance, therefore, patients were only asked to provide consent to treatment—to have blood cultures collected and receive antibiotics, if randomly allocated to the intervention arm.

Patients included in the feasibility trial were sent a Patient Information Leaflet, which included a ‘Participant Dissent Form’ at 90 days after the patient’s emergency call (30 days for those recruited in the last two months of the study), offering options to either decline to receive any further correspondence from the study or to have all of their records withdrawn from the study. We included public contributors in discussions about this approach which minimises intrusion and possible distress.

### Data collection

Routine ambulance service records were used for all prehospital information. Anonymised data relating to clinical outcomes and hospital diagnoses were obtained from hospital records as well as through the Secure Anonymised Information Linkage (SAIL) databank^18^. The SF-12 and Quality of Care Monitoring forms were sent out to participants at 90 days (30 days for participants recruited in the last two months of the study) along with the Patient Dissent Forms to be returned directly to the research team at Swansea University for data entry. Returned questionnaire responses were linked to the anonymised data for analysis within the SAIL gateway.

#### Adverse events

After consultation with the Trial Management Group (TMG) and Trial Steering Committee (TSC), it was decided that deaths and Intensive Care Unit (ICU) admissions were expected and would not be collected as adverse outcomes. Serious Adverse Reactions (SARs) to be monitored were agreed as:

- Anaphylaxis
- *C. difficile* infection
- Extravasation at the site administration of antibiotics
- Infection/cellulitis at the site of blood culture collection/administration of antibiotics
- Vascular damage at the site of blood culture collection/administration of antibiotics

#### Missed recruitments

As part of this feasibility study, we monitored missed recruitments in order to assess whether paramedics were able to accurately recognise patients with sepsis; whether paramedics were compliant with the protocol; and how many patients could not be randomised owing to exclusion criteria emergency.

### Progression criteria

At the outset of the feasibility trial, the Trial Management Group (TMG) specified progression criteria, to be met within reasonable limits; if a progression criterion was within 5% below target we would review reasons for this and consider modifications to protocol; if within 10% we would critically review reasons for this and assess whether major changes to protocol are likely to improve the issue; if more than 10% we should consider whether progression to full trial is appropriate.

### Intervention feasibility

1. *Compliance with protocol by paramedics—*>  = 80% of patients recognised as eligible by study paramedics are randomly allocated to trial arm
2. *Acceptability of intervention to patients*—mean patient satisfaction in intervention group > 80% in the control group
3. *Safety* – number of patients who experience adverse events has a difference of < 10% between trial arms
4. *Recognition of sepsis –*
  a. > 50% of patients with sepsis who are attended by study paramedics are recognised as eligible for the study
  b. > 70% of randomised patients are diagnosed with sepsis in hospital

### RCT Feasibility

5. *Acceptability of RCT to paramedics—*≥ 60% of eligible paramedics agree to take part in the study.
6. *Acceptability of RCT to patients* – dissent to take part in the study is ≤ 30%.
7. *Retrieval of outcomes*—follow up data for primary outcome can be collected for ≥ 70% of patients.
8. *Equipoise—*findings indicate that we remain in equipoise about the effectiveness of paramedic obtained blood cultures and prehospital antibiotics for sepsis.
9. *Recruitment*—recruitment target met to ≥ 80%.

### Sample size

Paramedics recruited patients over a six-month period. Based on data regarding throughput of sepsis cases in ED, we estimated recruitment of approximately 100–150 patients. In this feasibility study we did not aim to detect differences between the intervention and control groups in outcomes, but to include enough patients to assess findings against our progression criteria.

### Randomisation

A randomisation schedule with a 1:1 ratio of ‘intervention’ or ‘control’, stratified by paramedic was produced by a statistician not involved in the data collection, management or analysis of the data set. A unique set of scratchcards with allocation concealed was issued to each study paramedic and kept on their person during each working shift^19^. When the study paramedic identified an eligible patient, they were instructed to use the next sequential scratchcard from their set out of sight of the patient. The unique number shown on the scratchcard became the patient’s Study ID. The paramedic retained the scratchcard in order to store it with the randomisation log at the nurses’ station in the Emergency Department, so that the Paramedic Research Support Officer (PRSO) was able to monitor randomisation. Owing to the nature of the intervention, the outcome of randomisation could not be blinded to the paramedics, patients, PRSO or data manager. The allocation was concealed from the statistician and health economist until the code for analysis was finalised.

### Health economics

The health economic component of this feasibility study focused on establishing the main cost drivers by estimating the intervention implementation cost through discussions with the trial team and research paramedics, using standard unit costs for the antibiotics given. We considered a suitable framework to undertake a full cost-effectiveness analysis in a future trial.

### Analysis

We analysed study data to inform progression decision and trial planning: we analysed continuous outcomes (e.g. SF-12 Score; length of stay) by t tests, or non-parametric equivalents; we report mean and standard deviation, or median and interquartile range, along with 95% confidence intervals. We analysed binary outcomes (e.g. mortality, presence of sepsis) by chi-squared tests; and report proportions and 95% confidence intervals. We conducted exploratory analysis of potential primary outcomes to assess whether we had met progression criterion 8 (whether we remain in equipoise), and to help decide what might be the most appropriate primary outcome for a fully-powered trial. We report against each of our progression criteria to inform whether we should seek further funding for an appropriately powered trial and provide estimates for performing a power calculation for a fully-powered trial. Analysis was performed using SPSS Version 25 and reporting follows relevant CONSORT checklists^20,21^.

### Public and patient involvement

Public and patient representatives contributed to designing, delivering, overseeing and disseminating the study. We recruited people with experience of sepsis, as either patients, carers or family members of patients, to the Trial Management Group and Trial Steering Committee. We provided briefing sessions prior to all meetings.

### Changes after the study began

Owing to low response rates to study questionnaires, we submitted a substantial amendment to the Research Ethics Committee to allow us to conduct the questionnaire by telephone if the postal questionnaire had not been returned.

### Ethics approval

Ethical approval was granted by Wales Research Ethics Committee 4, reference 17/WA/0186.

## Results

**Figure 1:**
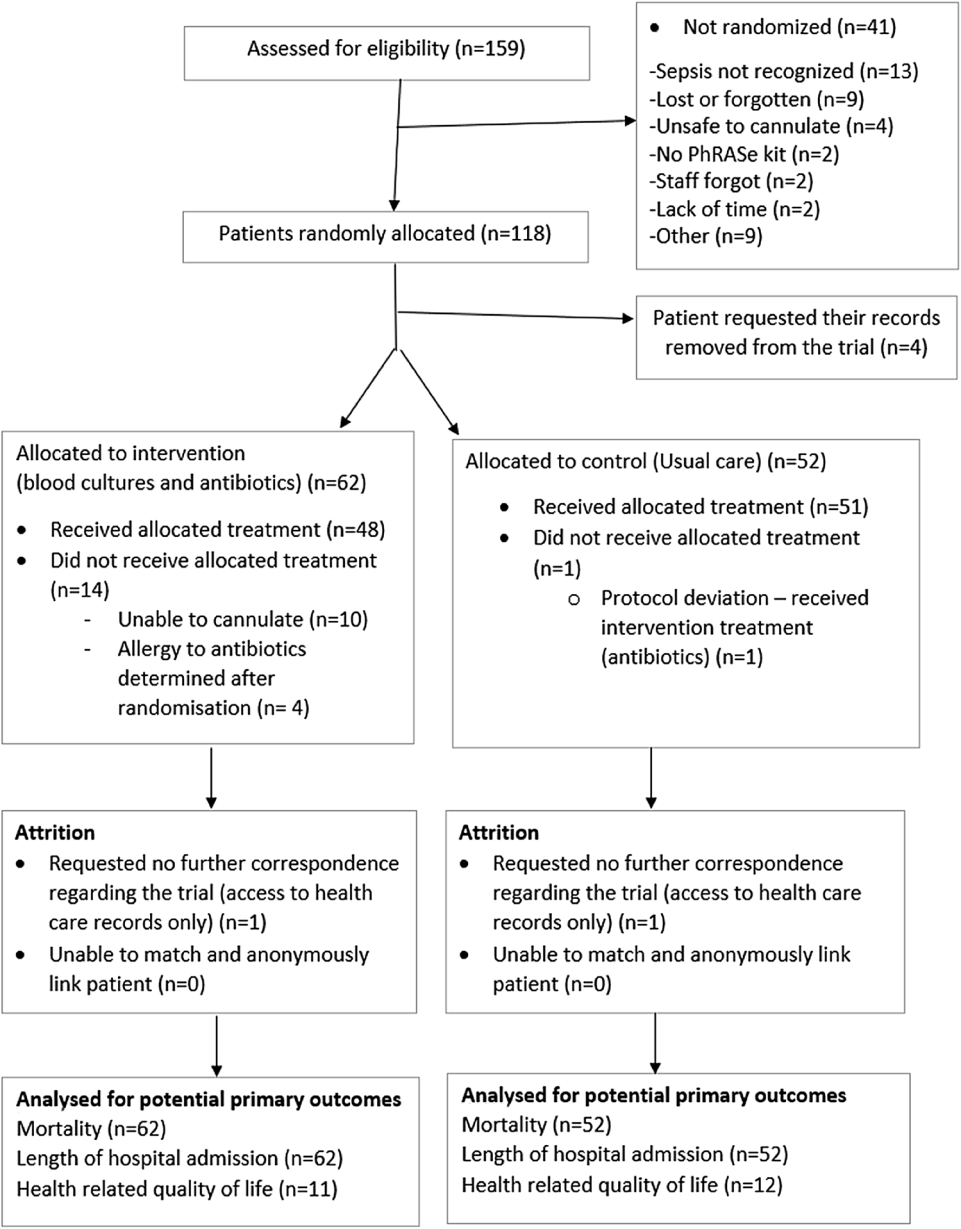
CONSORT flowchart.

We report feasibility study findings in accordance with relevant CONSORT and GRIPP 2-SF checklists^21,22^; the CONSORT flowchart is seen in Fig. 1.

### Baseline characteristics

Table 1 shows patients’ baseline characteristics. Sixty-two patients (54%) were allocated to the intervention arm. The mean age of the intervention arm was 75.6 years (range 30–99) and 38 patients (61%) were female. The mean age of the control arm was 71.2 years (range 28–97); 33 (65%) control participants were female. qSOFA scores (which predicts risk of in hospital mortality, with 3 being the highest risk) show that more patients had scores of 2 or 3 in the intervention group, though this wasn’t statistically significant. Nine patients in the control group (18%) and 17 in the intervention group (28%) were already taking antibiotics at the time of their emergency call.

**Table 1:** Baseline characteristics.

Characteristic	Intervention (n = 62)	Control (n = 52)
Age in years, mean (range)	75.6 (30–99)	71.2 (28–97)
Female, n (%)	38 (61%)	33 (65%)
**qSOFA score, n (%)**		
0	5 (8.1)	2 (3.8)
1	38 (61.3)	34 (65.4)
2	15 (24.2)	11 (21.2)
3	3 (4.8)	1(1.9)
N miss (%)	1 (1.6)	4 (7.7)
Already taking antibiotics at emergency call, n (%)	17 (28)	9 (18)

### Recruitment, consent and response rates

Seventy-four of the 104 (71.2%) eligible paramedics who worked in the locality at the start of the study agreed to take part, and 54 (73%) completed their training. The majority of paramedics recruited at least one patient to the trial (n = 39) with most of them recruiting <  = 3 patients (n = 26) and the maximum number of patients recruited by one paramedic being seven.

Patients were recruited from 1.12.17 to 31.5.18. In total, 118 patients were randomly allocated to the two trial arms; four patients subsequently dissented to be included in the trial, leaving 114 patients recruited to follow-up. Two patients (one from each arm of the trial) requested no further correspondence so we collected data from their medical records only. We matched 100% of patients to SAIL records. Twenty-three completed questionnaires were returned out of 82 (28%) sent to participants who had not died before 90 days (12 of 41 (29%) control and 11 of 41 (27%) intervention).

The diagnoses recorded in the ED notes showed 51 of the 114 (45%) recruited patients were diagnosed with sepsis. Thirty-nine of the 114 (34%) patients were recorded as having a non-viral infection, most commonly pneumonia or urinary tract infection. Thus, 90 out of the 114 (79%) recruited patients had sepsis or a likely bacterial infection recorded in ED. No serious adverse reactions were recorded.

### Accuracy of recognition of sepsis by paramedics

By assessing records from the ED of patients who were treated in the resuscitation department for suspected sepsis (as documented in the ED resuscitation log book), we recorded 41 patients as ‘missed recruitments’ (meaning there were 159 eligible patients in total). In 13 of the missed recruitments, the patient was not recognised as having sepsis. In the other 28 missed recruitments, who were recognised as having sepsis but not randomly allocated to the trial, reasons cited included lost or forgotten scratchcards (n = 9); no PhRASe kit on the vehicle (n = 2); lack of time (n = 2); forgot to enter in the study (n = 2); unsafe/unable to cannulate (n = 4); and ‘other’ (n = 9). This means that 118 of 159 total eligible patients were allocated to trial arms (74%), and 118 out of 146 patients recognised as eligible by the study paramedic were allocated to trial arms (81%).

### Protocol deviations

Eighteen protocol deviations were recorded (Table 2). All three patients who were initially not recorded in the randomisation log were identified from WAST clinical records and included in follow up. One patient was taken to a non-study hospital as a result of a hospital divert protocol being implemented during a period of high demand at the ED.

**Table 2:** Protocol deviations.

Deviation	n
Allergy to antibiotics identified after the patient was randomly allocated (no antibiotics given)	4
Lost or damaged scratchcards	4
Patient not recorded in randomisation log	3
Missing kit or component	2
Blood culture forms not completed	1
Incomplete dose of Cefotaxime administered	1
Scratchcards used out of order	1
Control patient given intervention	1
Patient taken to non-receiving hospital	1
Total	18

### Health economics

Intervention implementation costs included paramedic training costs and total callout costs (including antibiotics and blood cultures in the intervention group). Fifty-four paramedics were trained in two 1.5-h sessions (online and face-to-face training) at a total cost of £8,128.50. This amounts to £68.89 per trial participant (n = 118). Per-patient training costs are subject to economies of scale and maydecrease if the intervention is on a larger scale. Additionally, total callout costs (comprising staff cost based on time from callout to arrival at hospital, cost of cefotaxime 2 g for intravenous administration^16^ and laboratory cost of blood culture^17^ were £23.33 (95% CI: -2.71 to 49.36; p = 0.09) higher in the intervention group, based on the additional drug and culture costs and an on average 10.2 min (95% CI − 9.0 to 29.4; p = 0.11) longer job cycle time.

Healthcare resource use data linked through the SAIL databank were available for all participants and showed that the main cost drivers can be expected to be inpatient admissions and ICU stays (Table 1). There was evidence of significant missing SF-12 data with only 23 patients (20.18%) returning completed questionnaires at 90 days.

### Outcome analyses

Unadjusted comparisons (seen in Table 3) between deaths; ICU admissions; ED attendances in the three months following the initial emergency call; length of hospital admission; job cycle and on-scene times satisfaction with care; and health-related quality of life showed no differences at the P = 0.05 level of significance. The only statistically significant difference between trial arms was the number of hospital admissions in the three months following the initial emergency call (87 in the experimental arm compared with 56 in the usual care arm, p = 0.0002).

**Table 3:** Unadjusted analysis of outcomes between groups.

Outcome	Intervention (n = 62)	Control (n = 52)	Total (N = 114)	Difference (95% CI, Significance level)
**Routine data (SAIL)**				
90 day Mortality n(%)	21 (33.9)	11 (21.2)	32 (28.1)	Odds ratio 1.9 (0.82, 4.5) *p* = 0.13
ICU admissions (yes) (for primary emergency call), n (%)	5 (8.1)	1 (1.9)	6 (5.3)	Odds ratio: 4.5 (0.51, 39.6) *p* = 0.14
Number of ED attendances up to 90 days from emergency call (mean, SD of ED attendance per patient)	79 (1.3, 0.5)	76 (1.5, 0.7)	155 (1.4,0.6)	Mean Diff − 0.2 (− 0.41, 0.03), *p* = 0.1
Number of hospital admissions up to 90 days from emergency call (mean, SD admission per patient)	87 (3.5, 3.3)	56 (1.8,1.0)	143 (2.83, 2.7)	Mean Diff 1.8 (1.0, 2.5). *p* < 0.05
Bed days used up to 90 days from emergency call (mean (95%CI), median, sd)	14.2 (8.8,19.5); 5, 25.2	14.4 (7.3, 21.5); 4, 26.5	14.3 (10.0,18.5); 4, 25.6	− 0.3 (− 8.9, 8.5), *p* = 0.9
**Data collected by PRSO**				
Time interval (minutes) from emergency call to administration of antibiotic m, sd, nmiss(%)	131, 147, 15 (24.2)	NA	NA	NA
Job cycle time (minutes) from time of call to arrival at hospital m, sd, nmiss(%)	155, 132, 0	136, 87.5, 0	146, 114, 0	18.7 (− 23,9, 61.3) *p* = 0.2
On scene time (minutes) m, sd	77.2, 131	65.5, 86.9	71.9, 113	11.7 (− 30.5,53.9) *p* = 0.3
Blood Culture received	48	NA	NA	NA
Contamination of blood cultures		NA	NA	NA
Yes	1 (2.1)			
No	42 (87.5)			
Missing	0			
Not possible to identify	5 (10.4)			
**Patient reported outcome measures**				
Quality of care monitor score	n = 6 39.7, 5.6	n = 9 39.7, 8.3	n = 15 39.7, 6.6	Mean diff: 0, (− 7.8, 7.8), *p* = 1.0
SF-12 score (at 90 days)	n = 11	n = 12	n = 23	
Physical component score	31.6 (11.2)	35.3 (12.6)	33.5 (11.8)	3.6, (− 6.7,13.9), *p* = *0.5*
Mental component score Mean (SD)	41.8 (14.5)	46.5 (15.7)	44.3 (14.9)	4.7, (− 8.5,17.8) *p* = *0.5*

*M* mean, *med* median, *min* minimum, *max* maximum, *sd* standard deviation, *n miss* number of missing.

### Sample size and recruitment required for a full trial

We have calculated the sample size required to find a 5% difference in mortality between groups, using 90% power and 5% significance, a difference agreed by the research team including PPI representatives and clinicians as important. Based on a 90 day mortality rate of approximately 28% in this feasibility trial, 3200 patients are needed in analysis. If approximately 10% of participants dissent from anonymised follow up of routine records, and we are unable to match 1% of cases in routine health data sources, we will need to randomly allocate 3591 patients. Using the PhRASe rate of recruitment and data on sepsis related calls from potential collaborating ambulance services, we will need five sites to each recruit approximately 718 patients during a 24 month recruitment period.

### Results summary—Fulfilment of Progression Criteria

This feasibility study met all of its pre-determined progression criteria, as summarised in Table 4.

**Table 4:** Summary of results against progression criteria.

Progression criterion	Relevant result	Interpretation
1: Compliance with protocol by paramedics—no less than 80% of patients recognised as eligible patients by study paramedics are randomised	118 of 146 (81%) eligible patients were recruited and randomly allocated to trial arm	Criterion met
2: Acceptability of intervention to patients—mean patient satisfaction in intervention group is not less than 80% of patient satisfaction in the control group	There was no evidence of any difference between groups in patient satisfaction	Criterion met
3: Safety—number of patients who experience adverse events has a difference of less than 10% between trial arms	No adverse reactions were reported. There were no statistically significant differences between intervention and control groups in Serious Adverse Events (ICU admissions; deaths)	Criterion met
4: Recognition of sepsis—a. at least 50% of patients with suspected sepsis who are attended by study paramedics are recognised as eligible for the study	118 of 159 (74%) patients who were recorded as having sepsis in ED were allocated to trial arms	Criterion met
4: Recognition of sepsis—b. at least 70% of randomised patients are diagnosed with sepsis in hospital	Ninety of 114 (79%) recruited patients were recorded as having sepsis or a likely bacterial infection	Criterion met
5: Acceptability of RCT to paramedics—at least 60% of eligible paramedics agree to take part in the study	Seventy-four of 104 (71%) eligible paramedics who work in the Cardiff and Vale locality expressed an interest to take part in PhRASe. Fifty-four paramedics completed their training (51.9%)	Criterion met within reasonable limits
6: Acceptability of RCT to patients—dissent from taking part in the study is 30% or less	Dissent to take part in the study was 3% (4/118)	Criterion met
7: Retrieval of outcomes—follow up data for primary outcome suitable for fully-powered trial can be collected for 70% or more of patients	All recruited patients were matched to routine data outcomes in SAIL	Criterion met
8: Equipoise—findings indicate that we remain in equipoise about the effectiveness of paramedic obtained blood cultures and prehospital antibiotics for sepsis	Only one outcome (number of hospital admissions in three months following emergency call) was statistically significantly different between trial arms	Criterion met within reasonable limits
10. Recruitment—recruitment target met to at least 80%	We allocated 118 patients to trial arms, with 114 ultimately recruited. (Our aim was to recruit 100–150 patients)	Criterion met

## Discussion

The PhRASe feasibility study achieved all of its progression criteria within reasonable limits (Table 4).

With regard to acceptability of the RCT to paramedics, more paramedics initially expressed an interest in taking part than completed training. It is not known why this was; we would need to consider what the barriers to participation were in order to assess whether this could be improved upon in a definitive trial. Though this feasibility trial invited paramedics to volunteer, we would in future try to recruit all paramedics in order to meet the required sample size.

As there was a 13% absolute difference in 90-day mortality between trial arms, we recommend that in a definitive trial mortality is monitored continuously, and reported on a monthly basis, to the independent Data Monitoring Committee.

On four occasions, patients were entered into the trial and subsequently found to have an allergy to antibiotics. We would have to further emphasise in training for a definitive trial that the patient’s drug allergies should be checked prior to patient recruitment.

Although the recently published PHANTASi trial^23^ of prehospital antibiotics in the Netherlands found that nurses giving antibiotics in the ambulance did not lead to improved survival, there are factors which make it different to our proposed study. The PHANTASi trial included patients with all severities of sepsis; the overall mortality rate was just 8% at 28 days (compared to 28% at 90 days in this feasibility study). The study was therefore underpowered to detect a difference in mortality in only those patients with severe sepsis. Ambulances in the Netherlands are staffed by nurses with years of experience in treating critically ill patients and who have followed additional specialised training before applying to qualify as a registered ambulance nurse. This research team proposes to conduct a randomised trial for patients with more severe illness, in an EMS system staffed by paramedics rather than nurses.

Weinberger et al.^24^ discuss the difficulties in assessing time to first antibiotics in patients with sepsis, as patients contact emergency services (or their family doctor or out of hours services) at different time points in their illness. Though we accept this is true, as we plan to conduct an RCT, the range of time patients’ have waited before calling emergency services should not systematically vary between groups. Weinberger et al. also argue that patients with multiple comorbidities may not present with typical symptoms of sepsis, as would an otherwise healthy person, which could prolong their wait for antibiotics. Again, there is no reason to believe one randomised group would have significantly more unwell patients in it, but, for this reason, we would ensure comorbidities are included as covariates in the multivariate analysis of outcomes in the full RCT.

### Limitations

Patient quality of life data using the SF-12 questionnaire were only obtained from 23 of 82 eligible participants (28%). While this had to be expected considering the health status of the population in question, the lack of response to the SF-12 quality of life questionnaire would need to be addressed in order to undertake a cost-utility analysis. This may include investigating barriers for questionnaire completion and putting into place measure to improve return rate of outcome measures.

### Interpretation and conclusion

We compared trial arms only to confirm that we remain in equipoise about the clinical effectiveness of pre-hospital antibiotics for sepsis administered by paramedics. Hence, we treat observed differences in outcomes with caution in this feasibility trial.

This feasibility study met its pre-determined progression criteria; an application will therefore be prepared and submitted for funding for a fully-powered multi-centre randomised trial.

### Trial status

Complete and reported to the funder in June 2019.

## Supplementary Information

Supplementary Information.

## Data Availability

Study data is available upon request.

## Abbreviations

AE: Adverse event
ED: Emergency department
IV: Intravenous
NHS: National health service
PI: Principal investigator
PPI: Patient and public involvement
PRSO: Paramedic research support officer
RCT: Randomised controlled trial
SAE: SAE event
TMG: Trial management group
TSC: Trial steering committee
WAST: Welsh ambulance services NHS trust
