# Author Correction: Randomised feasibility study of prehospital recognition and antibiotics for emergency patients with sepsis (PhRASe)

**DOI:** 10.1038/s41598-022-06469-0

**Published:** 2022-02-03

**Authors:** Jenna Jones, Susan Allen, Jan Davies, Timothy Driscoll, Gemma Ellis, Greg Fegan, Theresa Foster, Nick Francis, Saiful Islam, Matt Morgan, Prabath W. B. Nanayakkara, Gavin D. Perkins, Alison Porter, Timothy Rainer, Samuel Ricketts, Bernadette Sewell, Tracy Shanahan, Fang Gao Smith, Michael A. Smyth, Helen Snooks, Chris Moore

**Affiliations:** 1 Swansea University Medical School, ILS2, Singleton Campus, Wales, SA2 8PP UK; 2 Cardiff and Vale University Health Board, Wales, UK; 3 Public Contributor, Wales, UK; 4 East of England Ambulance Service NHS Trust, Royston, England UK; 5 University of Southampton, Southampton, UK; 6 Amsterdam University Medical Centre, Amsterdam, The Netherlands; 7 University of Warwick, Warwick, England UK; 8 Welsh Ambulance Services NHS Trust, Wales, UK; 9 Swansea Centre for Health Economics, Swansea University, Wales, UK; 10 University of Birmingham, Birmingham, England UK

Correction to: *Scientific Reports* https://doi.org/10.1038/s41598-021-97979-w, published online 20 September 2021

The original version of this Article contained errors in Table 1 and Table 3.

In Table 1, in the group “Already taking antibiotics at emergency call, n (%)”, the number of patients in “Intervention (n = 62)” and “Control (n = 52)” were interchanged. The incorrect and correct values appear below.

Incorrect:

**Table:** 

Characteristic	Intervention (n = 62)	Control (n = 52)
**qSOFA score, n (%)**		
Already taking antibiotics at emergency call, n (%)	9 (18)	17 (28)

Correct:

**Table:** 

Characteristic	Intervention (n = 62)	Control (n = 52)
**qSOFA score, n (%)**		
Already taking antibiotics at emergency call, n (%)	17 (28)	9 (18)

In Table 3, the p value for the outcome “Number of hospital admissions up to 90 days from emergency call (mean, SD admission per patient)” was incorrect. The incorrect and correct values appear below.

Incorrect:

**Table:** 

Outcome	Intervention (n = 62)	Control (n = 52)	Total (N = 14)	Difference (95% CI, Significance level)
**Routine data (SAIL)**				
Number of hospital admissions up to 90 days from emergency call (mean, SD admission per patient)	87 (3.5, 3.3)	56 (1.8, 1.0)	143 (2.83, 2.7)	Mean Diff 1.8 (1.0, 2.5). *p* = 0.00

Correct:

**Table:** 

Outcome	Intervention (n = 62)	Control (n = 52)	Total (N = 14)	Difference (95% CI, Significance level)
**Routine data (SAIL)**				
Number of hospital admissions up to 90 days from emergency call (mean, SD admission per patient)	87 (3.5, 3.3)	56 (1.8, 1.0)	143 (2.83, 2.7)	Mean Diff 1.8 (1.0, 2.5). *p* < 0.05

The original Article has been corrected.

