# Supplementary material for: Randomised feasibility study of prehospital recognition and antibiotics for emergency patients with sepsis (PhRASe)
Source: Sci Rep. 2021 Sep 20;11:18586. doi: 10.1038/s41598-021-97979-w (PMC8452688; doi:10.1038/s41598-021-97979-w)
Supplement: Supplementary file 1 — Supplementary Information. [file 41598_2021_97979_MOESM1_ESM.pdf]

Ymddiriedolaeth GIG Gwasanaethau Ambiwlans Cymru
Welsh Ambulance Services NHS Trust
Ymchwil Iechyd a Gofal Cymru
Health and Care Research Wales
Ariennir gan Lywodraeth Cymru
Funded by Welsh Government
Swansea University Medical School
Ysgol Feddygaeth Prifysgol Abertawe

PhRASe
Prehospital Recognition and Antibiotics for 999 patients with Sepsis: a feasibility study

THE UK SEPSIS TRUST

PRE-HOSPITAL SEPSIS SCREENING TOOL

Is the NEWS 3 or above?

Does the patient look very sick?

NO → Low risk of sepsis. If concerned consider other diagnoses, transfer as required. Use standard protocols. If not for transfer, recommend contact GP/OOH/111 if concerned, or 999 if patient deteriorates rapidly

YES ↓

Is the history suggestive of infection?

- Yes, but source not obvious
- Pneumonia/ likely chest source
- Urinary Tract Infection
- Abdominal pain or distension
- Cellulitis/ septic arthritis/ infected wound
- Device-related infection
- Meningitis

NO → Low risk of sepsis (see above)

YES ↓

Are any Red Flags present?

- Responds only to voice or pain/ unresponsive
- Systolic B.P ≤ 90 mmHg (or drop >40 from normal)
- Heart rate > 130 per minute
- Respiratory rate ≥ 25 per minute
- Needs oxygen to keep SpO2 ≥92%
- Non-blanching rash, mottled/ ashen/ cyanotic
- Not passed urine in last 18 hours
- Recent chemotherapy

NO → Are any Amber Flags present?

YES ↓

Red Flag Sepsis!

Follow PhRASe Protocol (overleaf)

Resuscitation and Pre-alert

- Oxygen to maintain sats >94% (88% if COPD)
- 250ml boluses of sodium chloride (max 250mls if normotensive, max 2000ml if hypotensive)

Are any Amber Flags present?

- Relatives concerned about mental status
- Acute deterioration in functional stability
- Immunosuppressed
- Trauma/surgery/procedure in last 6 weeks
- Respiratory rate 21-24 OR breathing hard
- Systolic BP 91-100mmHg
- Heart rate 91-130 OR new arrhythmia
- Not passed urine in last 12-18 hours
- Temperature <36C
- Clinical signs of wound, device or skin infection

NO → Give safety netting advice: call 999 if patient deteriorates rapidly, or call 111/ arrange to see GP if condition fails to improve or gradually worsens. Signpost to available resources as appropriate.

YES ↓

Sepsis likely

Transfer to designated destination.

Communicate likelihood of sepsis at handover.

Ymddiriedolaeth GIG Gwasanaethau Ambiwlans Cymru
Welsh Ambulance Services NHS Trust
Ymchwil Iechyd a Gofal Cymru
Health and Care Research Wales
Ariennir gan Lywodraeth Cymru
Funded by Welsh Government
Swansea University Medical School
Ysgol Feddygaeth Prifysgol Abertawe
Swansea University
Prifysgol Abertawe

PhRASe

TRIAL PROTOCOL AIDE MEMOIRE

Patient identified as having Red Flag Sepsis

↓

Will the patient be conveyed to UHW?

- NO → Follow usual care. Patient NOT eligible for PhRASe
- YES ↓

Do any of the following exclusion criteria apply?:

- Under 18
- Pregnant
- Known allergy to antibiotics

- YES → Follow usual care. Patient NOT eligible for PhRASe
- NO ↓

Use next sequentially numbered scratchcard out of sight of the patient
NB keep the scratchcard to record outcome on randomisation log

- **CONTROL** → Offer usual care
- **INTERVENTION** ↓

CLINICAL PROTOCOL

INTERVENTION

1. Cannulate patient (→ Unable to cannulate after 3 attempts → Offer usual care; → Patient refusal)
2. Take blood cultures (→ Patient refusal)
3. Administer Cefotaxime 2g IV (→ Patient refusal)
4. Continue with usual care

Patient refusal → Offer usual care

↓

On way to ED:
Label blood culture bottles with interim labels around neck of bottle (do not cover barcode)
Fill in specimen request form including WAST incident number
NB if attending paramedic was working on RRV, they must swap to the ambulance to travel with the patient to ED themselves

↓

On arrival at ED:
Handover blood culture bottles and specimen request form separately
(they require further labelling)
Ensure triage nurse writes time of first dose of antibiotics on sepsis six sticker

↓

Replace used PhRASe Trial Box components from paramedic cupboard at ED Reseal with new dated cable tie

↓

Before leaving ED: (also follows from Offer usual care)
Complete randomisation log located in UHW resus and file scratchcard with it
Text the Research Support Officer (07387249874) the incident number
